# Direct conversion of mouse embryonic fibroblasts into functional keratinocytes through transient expression of pluripotency-related genes

**DOI:** 10.1186/s13287-016-0357-5

**Published:** 2016-07-29

**Authors:** Demetris Iacovides, Gizem Rizki, Georgios Lapathitis, Katerina Strati

**Affiliations:** 1 Department of Biological Sciences, University of Cyprus, Nicosia, Cyprus; 2 Transgenic Mouse Facility, Cyprus Institute of Neurology and Genetics, Nicosia, Cyprus; 3 Current address: Department of Biology, Massachusetts Institute of Technology, Cambridge, MA USA

**Keywords:** Direct reprogramming, Transdifferentiation, Keratinocytes

## Abstract

The insufficient ability of specialized cells such as neurons, cardiac myocytes, and epidermal cells to regenerate after tissue damage poses a great challenge to treat devastating injuries and ailments. Recent studies demonstrated that a diverse array of cell types can be directly derived from embryonic stem cells (ESCs), induced pluripotent stem cells (iPSCs), or somatic cells by combinations of specific factors. The use of iPSCs and direct somatic cell fate conversion, or transdifferentiation, holds great promise for regenerative medicine as these techniques may circumvent obstacles related to immunological rejection and ethical considerations. However, producing iPSC-derived keratinocytes requires a lengthy two-step process of initially generating iPSCs and subsequently differentiating into skin cells, thereby elevating the risk of cellular damage accumulation and tumor formation. In this study, we describe the reprogramming of mouse embryonic fibroblasts into functional keratinocytes via the transient expression of pluripotency factors coupled with directed differentiation. The isolation of an iPSC intermediate is dispensable when using this method. Cells derived with this approach, termed induced keratinocytes (iKCs), morphologically resemble primary keratinocytes. Furthermore they express keratinocyte-specific markers, downregulate mesenchymal markers as well as the pluripotency factors Oct4, Sox2, and Klf4, and they show important functional characteristics of primary keratinocytes. iKCs can be further differentiated by high calcium administration in vitro and are capable of regenerating a fully stratified epidermis in vivo. Efficient conversion of somatic cells into keratinocytes could have important implications for studying genetic skin diseases and designing regenerative therapies to ameliorate devastating skin conditions.

## Introduction

The ability to generate differentiated cells from pluripotent cells holds great promise for regenerative medicine. Recently emerged techniques to reprogram somatic cells into induced pluripotent stem cells (iPSCs) and to transdifferentiate cells into different fates offer unprecedented possibilities to repair or replace damaged tissues [32, 33, 37]. These techniques potentiate autologous cell replacement therapies, thus minimizing issues of immune rejection and ethical constraints posed by embryonic stem cell (ESC) isolation. Although ESCs and iPSCs possess tremendous differentiation potential, efficiency of differentiation, risk of tumor formation, and accumulation of DNA damage during in-culture maintenance pose challenges to utilizing these cells in translational applications. In vivo reprogramming, or direct lineage conversion, can minimize unnecessary ex vivo manipulation of cells thereby reducing the risk of damage accumulation during reprogramming [5].

Recent studies demonstrate the feasibility of generating functional cells within native tissues via transdifferentiation. For example, forced expression of critical cardiac transcription factors in mouse hearts can stimulate the regeneration of de novo cardiomyocyte-like cells from terminally differentiated fibroblasts after ischemic heart damage [25, 29]. Reprogramming strategies have also been invaluable in studying and replicating cell types, such as neurons, with limited regenerative potential. Using patient-derived iPSCs, neurological and psychiatric diseases, including Alzheimer’s and schizophrenia, have been modeled in culture, providing crucial insights into disease mechanisms [9, 17]. Furthermore, several types of neurons, including multipotent progenitors, can be derived from pluripotent or differentiated cell types and have been used in proof-of-principle cell replacement studies in mouse models with successful therapeutic outcomes [21, 24, 36, 38]. These studies open up previously unrecognized avenues for the application of reprogramming to treat dementia, Alzheimer’s, and other neurodegenerative diseases. While most of these coming-of-age technologies have advanced the fields of reprogramming and regenerative medicine, there is an urgent need for more efficient cellular regeneration and repair paradigms.

The skin is a tissue that is highly prone to external injury or cancer development and is easily accessible, making it amenable to cell replacement therapy. Currently, tissue grafting is the most common skin tissue replacement therapy for extensive burns, acute and chronic wounds, reconstructive surgery, and genetic conditions such as epidermolysis bullosa [34, 35]. Skin grafting, which requires transplantation of donor tissue onto the wound, is often restricted by the availability of donor skin. In recent years, the use of cultured epithelial autografts (CEAs) generated by isolating and expanding epidermal stem cells (keratinocytes) in culture have provided a more effective tool to generate sheets of autologous skin cells, cover wounds, and enhance healing [3]. However, the long culturing periods necessary to obtain keratinocyte sheets, surgical complications of engrafting CEAs, and variable success rates of wound closure represent serious shortcomings of this procedure. More recently, methodologies to differentiate keratinocytes either from pluripotent ESCs or iPSCs have been described, thus providing an alternate source of skin cells [2, 7, 8, 18]. In an effort to improve keratinocyte derivation and to circumvent the challenges posed by using ESCs or iPSCs, we established a new paradigm for the conversion of mouse fibroblasts to functional keratinocytes, bypassing the isolation of a pluripotent intermediate. Our approach has the potential to facilitate efficient and large-scale keratinocyte generation for biological research and future cell-based regenerative applications.

## Materials and methods

### Cell culture

iPSC media were prepared with Dulbecco’s modified Eagle’s medium (DMEM) supplemented with 75 mL (15 %) knockout serum replacement (KSR), 5 mL (1 %) 5000 U/ml penicillin/streptomycin (P/S), 5 mL 50 mM BME, and 5 mL MEM non-essential amino acids (Gibco, cat. #11140-050). Mouse embryonic fibroblast (MEF) media contained 1× DMEM, 10 % fetal bovine serum, and 1 % P/S. When necessary, media were also supplemented with 500 μL LIF (10^6^ units/ml; Millipore, cat. #ESG1106). Defined keratinocyte serum free (DKSF) media were obtained from CellnTec, and were supplemented with 5 mL P/S. For the attachment of keratinocytes, cell culture dishes were coated with fibronectin (50 μg/mL) and collagen (100 μg/mL) for 20 min at 37 °C before culture. Keratinocytes (KCs) were cultured in medium low in calcium (0.07 mM) to maintain an undifferentiated status, and were differentiated in medium with a high calcium concentration (0.35 mM) to induce differentiation.

### Transfection and transduction

Actively growing 293 T cells were transfected with pCL-Eco, pSox2, pOCT4, pKlf4, or pGFP plasmids at 70 % confluency using Fugene 6 transfection reagent (Roche, cat. #11988387001) according to the manufacturer’s protocol. Cell media were changed the next day, and supernatants containing retroviral vectors were collected 48-h post-transfection and filtered through a 45-μm filter. Polybrene (Milipore cat. #TR-1003) was added at 10 μg/mL and 1 mL from each transcription factor was then used to transduce MEFs that were previously passaged only once. Transduction was repeated four times with at least 6-h intervals between.

### RNA extraction and quantitative reverse transcription PCR

RNA was extracted from MEFs, primary keratinocytes, and induced keratinocytes (iKCs) using the RNeasy kit (Qiagen, cat. #74104), DNase-treated with TURBO DNA-free kit (Ambion, cat. #AM1907), and quantified using NanoDrop. cDNA synthesis was performed using the iScript DNA synthesis kit (Bio-Rad, cat. #170-8890); 100 ng cDNA from each sample was then used as a template for polymerase chain reaction (PCR) using KapaTaq polymerase (KAPA BIOSYSTEMS, cat. #KK1014) and primers specific to keratinocyte markers (K14, K5, K8, K1), fibroblast markers (vimentin), and actin. Primer sequences were as follows:

K14: ACCGCAAGGATGCTGAGGA (fwd), GAAATCTCACTCTTGCCGCTCTG (rev); K8: TGCAGAACATGAGCATTC (fwd), CAGAGGATTAGGGCTGAT (rev); K1: TCGTGACCATCAAGAAGGAT (fwd), ACAACATTGGTTTCGCTGAT (rev); K5: CAAATCGACCCCACCATCCA (fwd), GAACCGCACCTTGTCGATGA (rev); vimentin: GACCGCTTTGCCAACTACATC (fwd), AGCATCTCCTCCTGCAATTTCT (rev); p63: AACCCCAGCTCATTTCTCGT (fwd), GGCCCGGGTAATCTGTGTTGG (rev); total Oct-4: CCAATCAGCTTGGGCTAGAG (fwd), CTGGGAAAGGTGTCCCTGTA (rev); total Sox-2: ATGGGCTCTGTGGTCAAGTC (fwd), CCCTCCCAATTCCCTTGTAT (rev); total Klf-4: CTGAACAGCAGGGACTGTCA (fwd), GTGTGGGTGGCTGTTCTTTT (rev); endogenous Oct-4: TCTTTCCACCAGGCCCCCGGCTC (fwd), TGCGGGCGGACATGGGGAGATCC (rev); endogenous Sox-2: TAGAGCTAGACTCCGGGCGATGA (fwd), TTGCCTTAAACAAGACCACGAAA (rev); endogenous Klf-4: GCGAACTCACACAGGCGAGAAACC (fwd), TTAGGCTGTTCTTTTCCGGGGCCACGA (rev) (endogenous Oct-4, Sox-2 and Klf-4 primer sequences from [33]; actin: CATCCGTAAAGACCTCTATGCCAAC (fwd), ATGGAGCCACCGATCCACA (rev). For quantitation of reverse transcription (RT)-PCR, keratinocyte and fibroblast marker expression was normalized to actin. Experiments were performed in triplicates and statistical significance was determined using a *t* test.

### Isolation of cells

MEFs were obtained by crossing FVB-Tg (KRT14-cre) mice (NCI mouse repository) with R26-stop-EYFP mice (Jackson laboratory, stock #006148). MEFs were isolated as follows: The uterus of the pregnant mouse was removed at day 13.5 post-conception and collected in phosphate-buffered saline (PBS) + antibiotics. Embryos were transferred to a 10-cm dish with PBS + antibiotics, and heads and viscera were cut off. The remaining body of each embryo was then transferred into a 6-cm dish with 1 ml 0.1 % trypsin solution and chopped up well with a razor blade. The dish was then incubated in a 37 °C incubator for 20 min, and the cell mixture was subsequently pipetted up and down 15–20 times to disperse the suspension of cells, and left in the incubator for an additional 20 min. At the end of incubation, 15 ml DMEM + 10 % fetal calf serum was added to each dish, and cells were transferred to a T75 flask for culture.

Primary keratinocytes were isolated from 2- to 4-day-old newborn mice. Following euthanasia, mice were soaked in betadine solution for 5 min, followed by 3× washes with 70 % ethanol. Limbs, tail, and the small part of the snout were subsequently removed, and a longitudinal cut from the snout to the tail was performed to enable skin removal. Skin was then peeled off and stretched on a sterile surface (dermis side down) and floated in 0.25 % trypsin overnight at 4 °C. The next day, skin was transferred to a sterile surface, with the epidermis side down, and the dermis was peeled off. The remaining epidermis was then minced with a razor blade and placed in a Falcon tube with complete KC medium supplemented with 1.4 mM Ca^2+^ for 1 h at 37 °C on a stirrer to obtain a single cell suspension. The cell suspension was then filtered through a cell strainer, and cells were plated on culture dishes pre-coated with collagen and fibronectin.

### Immunofluorescence

Cells were plated on coverslips pre-coated with poly-l lysine for 5 min. Next day, they were fixed with 4 % paraformaldehyde for 15 min at room temperature, washed twice with PBS for 5 min, permeabilized with 0.5 % Triton-X for 10 min at room temperature and washed three times with PBS (5 min each time). Fixed cells were then incubated in PBG blocking buffer (0.2 % cold water fish gelatin (SIGMA G-7765), 0.5 % bovine serum albumin (SIGMA A-2153) in PBS) for 30 min at room temperature, and incubated in PBG plus primary antibody for 2 h at room temperature, and washed twice with PBG for 5 min, followed by 45-min incubation in PBG plus FITC-labeled anti-rabbit secondary antibody (Jackson, cat. #715-095-150). Nuclei were stained with 100 ng/mL DAPI stain in PBG, followed by 2× washes with PBS for 5 min. Coverslips were then placed on slides with mounting medium (Vector Labs, cat. #H-1000) and viewed under a fluorescent microscope. Primary antibodies used were rabbit polyclonal to vimentin (Abcam, cat. #ab45939) and rabbit polyclonal to keratin 14 (Covance, cat. #PRB-155P).

### Western blot analysis

Cells were lysed in RIPA buffer, and protein concentration was determined with the Bio-Rad Protein assay using protein assay dye reagent concentrate (Bio-Rad, cat. #500-0006). Total lysates were separated on a 10 % SDS-PAGE gel for 1 h at 200 V. Proteins were subsequently transferred to a nitrocellulose membrane via semi-transfer for 25 min at 20 V, blocked with 5 % milk in TBS-Tween for 1 h at room temperature, and then incubated with the appropriate primary antibody at 4 °C overnight. Primary antibodies used were: rabbit polyclonal to vimentin (Abcam, cat. #ab45939), rabbit polyclonal to keratin 14 (Covance, cat. #PRB-155P), and rabbit polyclonal to actin (Abcam, cat. #ab8226). Membranes were then incubated in blocking buffer plus anti-rabbit HRP-conjugated secondary antibody (Santa Cruz, cat. #SC2054) for 1 h at room temperature and exposed using the LumiSensor™ chemiluminescent HRP substrate kit (GenScript, cat. #L00221V500).

### Tissue fixation, embedding, and staining

Skin removed from transplanted nude mice was fixed in 4 % freshly prepared paraformaldehyde overnight at 4 °C, and paraffin-embedded according to standard protocols. Paraffin-embedded tissues were sectioned into 10-μm sections, placed on glass slides, deparaffinized with xylene, and rehydrated in sequentially decreasing concentrations of ethanol. Slides were subsequently rinsed in PBS (2 × 3 min), microwaved at 130 °C for 10 min for antigen retrieval, and rinsed again with PBS (2 × 3 min). Tissue sections were blocked in 5 % horse serum in PBS for 2 h at room temperature, and treated overnight at 4 °C with the appropriate primary antibody diluted in block buffer. To visualize yellow fluorescent protein (YFP)-positive cells in epidermal tissue, we used an antibody against green fluorescent protein (GFP; Abcam, cat. #Ab290) which can recognize YFP, since this protein is a GFP point mutant. Primary antibodies used were anti-K14 at 1:1000 (Covance, cat. #PRB-155P), anti-K10 at 1:250 (Santa Cruz Biotechnology, cat. #sc-23877), and anti-loricrin at 1:250 (Covance, cat. #PRB-145P). Slides were then rinsed with PBS (2 × 3 min) and incubated for 45 min at room temperature in FITC-labeled (Jackson, cat. #715-095-150) or Cy3-labeled secondary antibody (Jackson, cat. #715-165-150) diluted 1:250 in block buffer. Samples were then mounted with mounting medium (Vector Labs, cat. #H-1000) and viewed under a fluorescent microscope. For hematoxylin and eosin (H&E) staining, tissue samples on glass slides were deparaffinized in xylene (2 × 10 min), rehydrated in sequentially decreasing concentrations of methanol, and subsequently stained with hematoxylin solution for 5 min, rinsed in tap water and placed in 5 % HCl/70 % EtOH solution for 30 s, followed by another rinse in tap water and incubation in eosin solution for 3 min. Slides were then rinsed off in tap water and tissues were dehydrated in increasing concentrations of methanol, incubated in 100 % ethanol for 5 min followed by 10 min incubation in xylene, and mounted using VectaMount permanent mounting medium (Vector Labs, cat. #H-5000).

### Cell grafting in nude mice

For cell transplantation experiments, athymic nude mice were used as previously described [20]. Briefly, mice were sedated with tribromoethanol (Sigma-Aldrich, cat. #T48402) at 0.2 mL per 10 g body weight. The backs of animals were decontaminated with betadine and 70 % ethanol. Using fine forceps, the full back skin was lifted and cut with scissors creating a graft area with a 1-cm diameter. A dome flange (Renner, cat. #F2 U 30268) was then inserted under the skin and tucked safely in place with wound clips. Cell suspensions were then applied to the graft area using a pipette through the hole on the top part of the dome flange. This study was carried out in strict accordance with the recommendations in the Guidelines for the Protection of Laboratory Animals of the Republic of Cyprus. The protocol was approved by the Veterinary Services (Republic of Cyprus Ministry of Agriculture, Rural Development and Environment; License number CY/EXP/PR.L1/2013). All euthanasia was performed by adhering to appropriate guidelines using a CO_2_ chamber, and all efforts were made to minimize suffering.

## Results

### Generation of induced keratinocytes from mouse embryonic fibroblasts

**Fig. 1:**
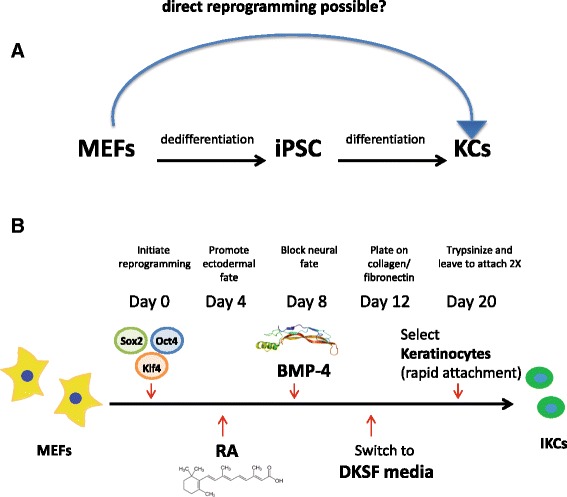
Conversion of mouse embryonic fibroblasts (*MEFs*) to functional keratinocytes (*KCs*), without isolating an induced pluripotent stem cell (*iPSC*) intermediate. **a** The hypothesis behind the experimental design. **b** After a brief initiation of pluripotency-inducing transcriptional reprogramming by transduction with Sox2, Oct4, and Klf4, induced MEFs are subsequently pushed towards the epidermal lineage by retinoic acid (*RA*) and bone morphogenetic protein-4 (*BMP-4*) treatment. *DKSF* defined keratinocyte serum free, *IKC* induced keratinocyte

Prior publications have described methods to differentiate mouse- or human-derived iPSCs or ESCs into epidermal keratinocytes in vitro by sequential administration of differentiation factors comprising retinoic acid (RA) and bone morphogenetic protein (BMP) [7, 8, 18]. Although direct lineage conversion between somatic cells of different types has been reported in the literature [36], generation of skin cells via direct reprogramming has not been demonstrated. To improve current keratinocyte-derivation technologies involving an iPS intermediate, we attempted to obtain keratinocytes directly from MEFs in vitro (Fig. 1a). We reasoned that a brief initiation of pluripotency-inducing transcriptional reprogramming mechanisms followed by directed differentiation towards the epidermal lineage may promote fibroblasts to switch towards the ectodermal fate, without fully committing to an iPS state. Recently, it was shown that such transient induction of pluripotency-related genes may be a viable option to transdifferentiate MEFs into other cell types via a non-isolated plastic state in order to differentiate in a directed manner [5, 19, 22]. Importantly, while prolonged systemic activation of these factors in vivo was previously shown to lead to teratoma formation [1], transient and/or localized delivery was not associated with adverse effects [22] even if c-Myc was included in the reprogramming mix [11, 39].

To this end, MEFs isolated from 2-day-old newborn mice and passaged only once were transduced with retroviral vectors expressing Sox2, Oct4, and Klf4 pluripotency-associated factors as previously described [33]. We repeated transduction twice a day for 2 days and the day of the fourth transduction was designated as day 0 of the experiment. The next day (day 1) we switched cell culture media to iPSC/ESC media and cells were left in culture for 4 days to allow for the expression of the transduced pluripotency factors and ensure enough time for initiation of pluripotency-related transcriptional reprogramming, which was previously shown to be initiated as early as 3 days after Sox2, Oct4, and Klf4 expression [30]. To promote the ectodermal fate we exposed cells to 0.3 μg/mL RA in iPSC/ESC media without LIF on day 4 [4] and continued culturing in RA until day 8, at which time it was removed. To block progression into the neural fate [13], cells were subsequently treated with 50 ng/mL BMP-4 and cultured for an additional 4 days. On day 12, cells were trypsinized and plated onto collagen/fibronectin-coated dishes, cultured in DKSF media and allowed to grow until day 20, at which time they were expanded into 60-mm dishes as described previously [7]. Since undifferentiated epidermal stem cells have been shown to rapidly attach collagen-coated surfaces, unlike differentiated skin cells [6, 7], RA/BMP-4 treated cultures were trypsinized, plated on collagen/fibronectin-coated dishes and allowed to attach for 15 min to enrich for keratinocytes. Unattached cells were washed off with PBS and the remaining population was cultured in DKSF medium until further expansion was necessary. We repeated the rapid-attachment procedure for two more passage cycles and subsequently harvested induced keratinocytes for additional experiments or froze the cells down in DKSFM + 10 % DMSO for future use (Fig. 1b).

**Fig. 2:**
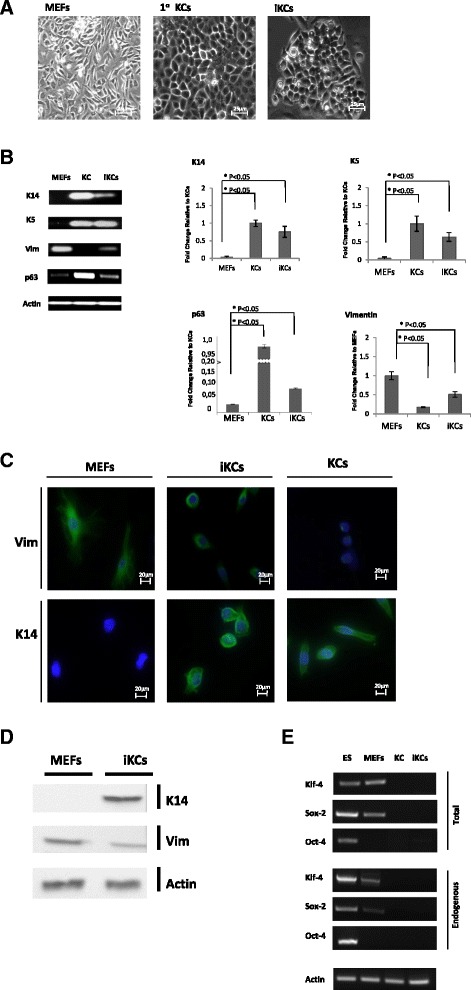
Morphological and molecular characterization of induced keratinocytes. **a** Induced keratinocytes (*iKCs*) exhibit keratinocyte-like morphological properties, closely resembling primary keratinocytes (*1°KCs*) in culture. iKCs displayed a round shape and formed colonies with a cobblestone-like appearance, a characteristic of primary keratinocytes in culture. In contrast, parental mouse embryonic fibroblasts (*MEFs*) exhibit mesenchymal characteristics, as they appear spindle shaped and do not form colonies. *Scale bar*s = 25 μm. (**b**) Expression levels of keratinocyte and fibroblast markers were measured by semi-quantitative RT-PCR, and agarose gel electrophoresis showing the product of each PCR reaction is depicted. The relative levels of each product from three independent experiments were normalized to actin. Results are depicted as fold-change of expression compared to expression in either primary keratinocytes (K14, K5, and p63) or in MEFs (vimentin). Error bars represent standard error between experiments. In contrast to MEFs, iKCs expressed undifferentiated keratinocyte markers (K14 and K5) at levels comparable to those of primary keratinocytes; p63 expression was threefold higher in iKCs compared to MEFs, even though KCs expressed p63 at much higher levels; vimentin expression was reduced twofold in iKCs, albeit not completely abolished. **c** Immunofluorescence staining against vimentin (*Vim*) and keratin 14 (*K14*) in MEFs, primary KCs, and iKCs. DAPI staining is shown in *blue*, and Vim or K14 staining is shown in *green. Scale bars* = 20 μm. (**d**) Western blot analysis to measure vimentin and K14 protein levels in MEFs and iKCs. Total cellular protein lysates were run and blotted with anti-vimentin and anti-K14 antibodies, as well as with anti-actin as a control. **e** Endogenous and total expression levels of pluripotency factors Klf4, Sox2, and Oct4 were assessed by RT-PCR in MEFs, KCs, and iKCs. ESCs were included as a positive control. Expression of these factors is silenced in iKCs

The cells obtained using this approach, which will henceforth be referred to as induced keratinocytes (iKCs), looked markedly different from MEFs and exhibited keratinocyte-like properties. In contrast to MEFs, iKCs appeared morphologically identical to primary keratinocytes in culture, displaying a rounded cellular morphology and forming colonies with a cobblestone-like appearance, a hallmark of primary keratinocytes in culture (Fig. 2a). MEFs mock-infected with control retrovirus constructs and incubated with RA/BMP-4 and DKSF media did not give rise to iKCs (data not shown), confirming that the derivation of keratinocytes depended on our transdifferentiation protocol and was not due to contaminating keratinocytes in the MEF preparations.

### Molecular characterization of induced keratinocytes

To ensure that the iKCs that displayed keratinocyte-like morphology were indeed skin stem cells, we further characterized iKCs at a molecular level. Using semi-quantitative RT-PCR we next measured the expression levels of keratinocyte and fibroblast markers in iKCs. In contrast to MEFs, iKCs expressed the undifferentiated keratinocyte markers keratin14 (K14) and keratin5 (K5) at levels comparable to those of primary keratinocytes (Fig. 2b). iKCs also expressed p63, an important gene in the commitment to keratinocyte fate, but at a level lower than that seen in primary keratinocytes. Even though the expression of vimentin, a fibroblast marker, was not completely abolished in iKCs, it was reduced by twofold compared to MEFs (Fig. 2b). These results were further confirmed using immunofluorescence staining and microscopy (Fig. 2c) as well as Western blotting (Fig. 2d). iKCs showed reduced expression of cytoplasmic vimentin, and exhibited a more round morphology than their parental MEFs. In contrast, MEFs expressed high levels of vimentin, which was clearly present in the cytoskeleton of these mesenchymal cells as expected. Similarly, iKCs expressed K14 protein, in contrast to the parental MEFs (Fig. 2c and d). Overall, our molecular experiments confirmed that the iKCs express characteristic keratinocyte-specific genes and begin to repress fibroblast markers, thereby resembling normal isolated keratinocytes. Importantly, expression of the reprogramming factors Oct4, Sox2, and Klf4 is silenced in the reprogrammed cells (Fig. 2e). This was assessed using primers for both endogenous and total levels of the factors.

### Functional characterization of induced keratinocytes

**Fig. 3:**
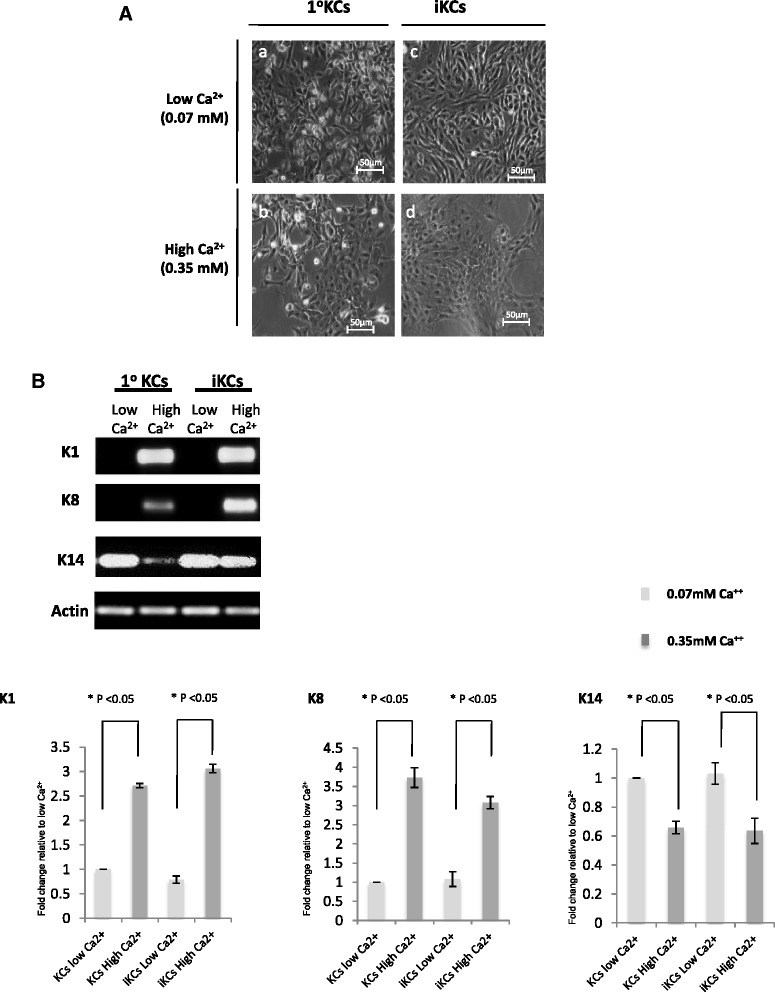
Induced keratinocytes (*iKCs*) can differentiate in vitro. (**a**) To test their differentiation potential in vitro, iKCs and primary keratinocytes (*1°KCs*) as a control were cultured under high (0.35 mM) and low (0.07 mM) Ca^2+^ concentrations. Primary KCs cultured in high calcium flattened out and differentiated (*b*), in contrast to cells cultured in low calcium which remained round and undifferentiated (*a*). iKCs recapitulated characteristics of primary keratinocytes under these conditions, and extensive differentiation was evident after an increase in Ca^2+^ levels (*c* and *d*). *Scale bars* = 50 μm. (**b**) Expression of the differentiation markers K1 and K8, as well as expression of K14, a marker of undifferentiated keratinocytes, were measured by RT-PCR under low or high Ca^2+^ culture conditions, and relative levels of each transcript from three independent experiments were quantified as before. Error bars represent standard error between experiments. Similar to primary keratinocytes, iKCs cultured under high Ca^2+^ conditions upregulated the expression of K1 and K8 and downregulated expression of K14 in response to elevated Ca^2+^ levels

Multipotent primary mouse keratinocytes have the capacity to differentiate in response to elevated extracellular calcium (Ca^2+^) concentrations. High calcium levels induce the expression of intermediate and terminal differentiation markers, such as K1 and K8, respectively, and result in stratification of keratinocytes [40]. In order to further characterize our iKCs in vitro, we next tested their differentiation potential under high Ca^2+^ conditions. Indeed, primary KCs cultured under a high Ca^2+^ concentration (0.35 mM) flattened out and appeared stratified, in contrast to cells cultured in low calcium (0.07 mM), which were round and unstratified (Fig. 3a, panels *a* and *b*). iKCs recapitulated characteristics of primary keratinocytes under these conditions, where extensive stratification was evident after an increase in Ca^2+^ levels (Fig. 3a, panels *c* and *d*). Cells cultured under high Ca^2+^ conditions upregulated the expression of the differentiation markers K1 and K8, unlike those cultured in low Ca^2+^ media (Fig. 3b). In addition, expression of K14, a marker of undifferentiated keratinocytes, was reduced in iKCs upon elevated Ca^2+^ levels and this reduction was comparable to that observed in primary keratinocytes (about 30 %) (Fig. 3b). These results suggest that iKCs derived from reprogrammed MEFs can be maintained in an undifferentiated state in vitro, are capable of differentiating in response to extracellular signals, and express differentiation markers similar to primary keratinocytes.

**Fig. 4:**
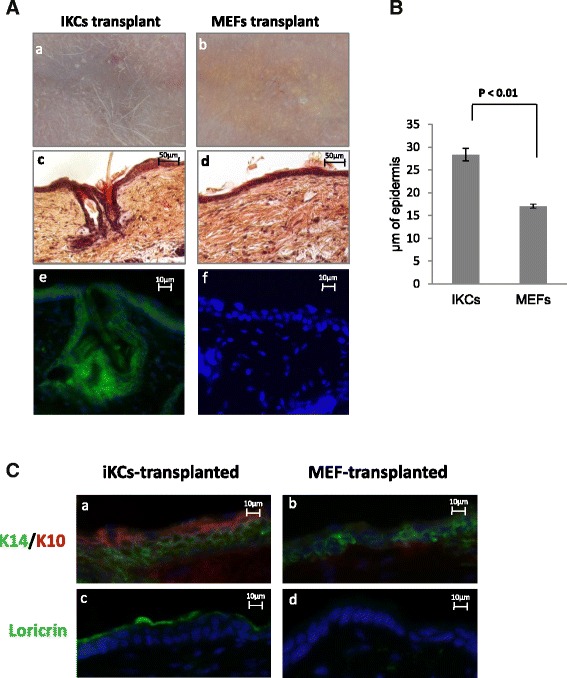
Induced keratinocytes (*iKCs*) can regenerate normal skin in vivo*.* (**a**) iKCs or mouse embryonic fibroblasts (*MEFs*) were transplanted in the back skin of nude mice. Nude mice transplanted with iKCs showed de novo hair formation at the graft site after 6 weeks (*a*), whereas hair development was not observed on mice transplanted with MEFs (*b*). All layers of the epidermis, including de novo hair follicles and sebaceous glands, are apparent in H&E stained skin sections from iKC-transplanted mice (*c*) but not MEF-grafted skin (*d*). EYFP staining (*green*) marks iKCs and de novo iKC-generated tissue (*e*), which is not present in MEF-transplanted epidermis (*f*). *Scale bars* = 50 μm (*c* and *d*) and 10 μm (*e* and *f*). (**b**) Epidermal thickness comparing iKC-derived vs MEF-transplanted skin. Blindfolded comparison was performed by measuring epidermal thickness in 25 images (two measurements/image) captured from five independent tissue sections (five images/section) from iKC-derived or MEF-derived skin. Note a nearly twofold increase in iKC-generated epidermis. Error bars represent standard error amongst 50 independent measurements for each sample. (**c**) Immunofluorescence staining for K14 (a marker of undifferentiated keratinocytes), K10 (an intermediate differentiation marker), and loricrin (a terminal differentiation marker). Tissue sections were stained for different keratinocyte stratification/differentiation markers in iKC- or MEF-transplanted tissue sections. IKC-transplanted epidermis expressed both K14 (*green*) and K10 (*red*), whereas MEF-transplanted tissue only displayed some K14-positive cells, and no K10 staining (*b*). Loricrin (*red*) was expressed on the outer epidermal layer of the iKC-derived skin (*c*) and indicates the presence of iKC-derived terminally differentiated cells. In contrast, MEF-derived skin was negative for loricrin expression (*d*). *Scale bars* = 10 μm

Undifferentiated keratinocytes possess the ability to regenerate normal skin and hair when grafted on nude mouse skins. To test whether iKCs were multipotent and thereby could differentiate and regenerate normal skin in vivo, we performed in vivo grafting in nude mice as described previously [20] and detailed in the Materials and methods section above. iKCs or MEFs were transplanted into chambers implanted under the back skin of nude mice. Approximately 6 weeks after grafting, nude mice transplanted with iKCs showed de novo hair formation at the graft site, whereas hair development was not observed in mice transplanted with MEFs (Fig. 4a, panels *a* and *b*). Mice were subsequently sacrificed and the skin from the graft area was collected for histological examination. iKCs were able to regenerate all layers of the epidermis, notably de novo hair follicles and sebaceous glands (Fig. 4a, panel *c*). In contrast, epidermal development of the MEF-grafted skin sections appeared incomplete, with no hair development and a thinner epidermis (Fig. 4a, panel *d*). When we measured the epidermal thickness of skin samples from both transplantation conditions, we indeed observed that the epidermis generated by differentiated iKCs was nearly twofold thicker than the epidermis generated at the MEF-transplanted site (Fig. 4b). The presence of hair growth, hair follicles, and sebaceous glands in iKC grafts, but not MEF-grafted skins, provides further evidence that iKCs are functional and can differentiate to give rise to different epidermal layers in vivo.

We originally used MEFs isolated from F1 mice derived from an R26-stop-EYFP × KRT14-cre cross, which could be used as a reporter to monitor K14 expression in cells. We hypothesized that the de novo epidermal tissue generated by iKCs should be positive for EYFP if the differentiated cells arose from the transplanted iKCs. In order to trace the origin of the cells contributing to epidermal regeneration, we used an antibody against GFP to detect EYFP levels by immunofluorescence. Indeed, EYFP staining was observed across the full epidermis of the iKC-transplanted animals, including all epidermal layers, the hair follicle, and the bulge (Fig. 4a, panel *e*). Conversely, the grafts from MEF-transplanted skins did not exhibit any EYFP staining, suggesting that the minimal epidermal regeneration that occurred in these animals was possibly due to keratinocytes in the host (Fig. 4a, panel *f*).

We next measured the expression of specific markers of various epidermal layers in the normal epidermis to determine whether the newly formed epidermis was correctly stratified. The undifferentiated marker K14 was expressed in the basal layer of the grafts in both the MEF-transplanted and iKC-transplanted mice (Fig. 4c, panels *a* and *b*). K10, a marker of intermediate differentiation, was detected at high levels in the layer immediately adjacent to the K14-positive stratum in iKC tissues but was barely detectable in the epidermis derived from the MEF-transplanted site (Fig. 4c, panel *b*). Finally, loricrin that is normally expressed in differentiated epithelium was observed on the outer epidermal layer of the iKC-derived skins. In contrast, MEF grafts did not display any loricrin staining (Fig. 4c, panels *c* and *d*). Collectively, these results indicate that keratinocytes obtained by transdifferentiation of MEFs in vitro have the capacity to reconstitute the fully stratified epidermis when transplanted onto nude mice.

## Conclusion

A critical limitation to cell replacement therapeutics is the procurement of good quality cells, in large enough numbers, with low chance of immune rejection. In the case of treatment of wounds caused either by physical causes, such as burns, or by disease, such as diabetic ulcers, cell replacement therapies have been extensively used [34, 35]. Keratinocytes which can be used as grafts or as a component of other more complex matrices to enhance re-epithelialization of a wound are a limited resource [14]. Derivation of keratinocytes from ESCs or iPSCs has been described as a way to generate larger numbers of these cells for cell replacement therapies or disease modeling [2, 7, 8, 18]. Recently, the transient expression of pluripotency-related factors followed by directed differentiation [5, 22] has been proposed as a potentially superior way to derive reprogrammed differentiated cells. This approach, as well as other transdifferentiation approaches, minimize ex vivo manipulation of cells without necessarily compromising safety [22, 39].

We describe in this manuscript a novel methodology for the direct conversion of mouse fibroblasts into induced keratinocytes. This methodology eliminates many intermediate steps necessary to generate iPSCs as a prerequisite, and has the potential for future adaptation in the native tissue. Molecular as well as in vitro and in vivo functional validation confirms the fidelity of these reprogrammed cells to primary keratinocytes. iKCs downregulate the expression of key mesenchymal markers, such as vimentin, and display elevated expression of several keratinocyte-specific genes, such as keratins. It has been previously reported that reprogrammed cells do not reach the full spectrum of transcriptional or other characteristics of the desired cell population [23]. Often, further culturing of the cells or other reprogramming modifications may lead to higher expression fidelity. It is important to note that cell functionality does not necessarily correlate with exactly similar transcriptional profiles. Furthermore, some transcriptome variation also exists among populations of the same type of cells [26]. A testament to their functionality, iKCs possess the ability to differentiate in response to increased calcium concentrations, and can regenerate skin, hair follicles, and sebaceous glands when transplanted in the backs of nude mice. Importantly, iKCs no longer express the reprogramming factors, which is a critical consideration for their safety in future in vivo applications.

Several improvements can contribute to the optimization of this technology towards the large-scale derivation of induced human keratinocytes for use in in vitro disease models or cell replacement studies, and potentially in vivo wound-healing approaches. Use of safer vectors, improvement of efficiency of reprogramming, and overall fidelity of generated cells to the natural cell type are a few examples of issues that generally apply to the majority of current reprogramming technologies. Efficiency of reprogramming and fidelity to the natural cell types are affected in part by intrinsic properties of the starting cells. For example, there are several reports suggesting that proliferation is essential for reprogramming and that a higher proliferation in the starting cell population correlates with higher reprogramming efficiency [27]. Nevertheless, excessive cellular proliferation associated with an upregulation of the growth factor signaling pathway has been reported to have an adverse effect on reprogramming [15].

It is of interest that the iKCs reported here seem to express keratinocyte-specific markers at levels slightly lower than keratinocytes, and fibroblast-specific markers at slightly higher levels. Small changes in culture conditions or changes as simple as prolonged culturing have previously been shown to improve the desired cell state, particularly in the case of iPS generation [23, 28, 31]. However it should be emphasized that the minor molecular differences described here do not place a barrier to cell function in vivo or in vitro. The degree to which reprogrammed cells should be similar to naturally occurring cells in order to be considered equally functional is very much under debate [12]. For example, in another study reporting the reprogramming of various human cells with the use of p63 and KLF4, the resulting cells have a high transcriptional fidelity to keratinocytes. However, they fail to perform in important functional tests, perhaps due to the fact that they express differentiated markers [10]. Although it is, of course, informative to assess the degree of cellular conversion based on transcriptional resemblance to the target cell’s identity, the ultimate determination of proper lineage conversion would likely depend on functional testing.

Our methodology employs the cocktail of transcription factors typically used in the derivation of iPSCs from transcription factors; however, this switches to directed differentiation culture conditions shortly after viral transduction. Using the methodology reported here we found that the reprogrammed iKCs no longer express any of the pluripotency genes used for reprogramming. Technologies such as the one described here validate earlier molecular data, which suggest that molecular hallmarks of “stemness” arise in reprogramming cultures long before iPS colonies are visible, typically as early as 4 days post-expression of pluripotency-promoting transcription factors [30]. Transient expression of reprogramming factors has recently been described in vivo and is not associated with the oncogenic effects seen in models where expression is sustained over longer periods, even if c-Myc is included in the reprogramming factors [39]. In addition to the molecular hallmarks of pluripotency in the early stages of reprogramming, functional pluripotency is also achieved, offering significant opportunities for manipulating the desired cell state. While the isolation of an iPSC with higher fidelity to ESC may require longer periods of culturing, earlier intermediates may be malleable enough for directed differentiation. Such intermediates have previously been isolated and described (dubbed pre-IPS or PiPS) [16, 22]. While they may lack key functions of ESCs and are inappropriate for some research uses, they may still be useful in achieving distinct differentiated cell states. Importantly, in contrast to iPS, they have been shown not to form teratomas in vivo [22]. Understanding the key steps in direct lineage-lineage conversions will enable the transfer of the technologies in vivo as demonstrated in cardiomyocyte studies [25, 29].

## Abbreviations

BMP, bone morphogenetic protein; CEA, cultured epithelial autograft; DKSF, defined keratinocyte serum free; DMEM, Dulbecco’s modified Eagle’s medium; ESC, embryonic stem cell; GFP, green fluorescent protein; iKC, induced keratinocyte; iPSC, induced pluripotent stem cell; KC, keratinocyte; MEF, mouse embryonic fibroblast; PBS, phosphate-buffered saline; PCR, polymerase chain reaction; RA, retinoic acid; RT-PCR, reverse transcription polymerase chain reaction; P/S, penicillin/streptomycin; YFP, yellow fluorescent protein

## References

[1] Abad M, Mosteiro L, Pantoja C, Canamero M, Rayon T, Ors I (2013). Reprogramming in vivo produces teratomas and iPS cells with totipotency features. Nature.

[2] Aberdam D (2004). Derivation of keratinocyte progenitor cells and skin formation from embryonic stem cells. Int J Dev Biol..

[3] Atiyeh BS, Costagliola M (2007). Cultured epithelial autograft (CEA) in burn treatment: three decades later. Burns..

[4] Bain G, Ray WJ, Yao M, Gottlieb DI (1996). Retinoic acid promotes neural and represses mesodermal gene expression in mouse embryonic stem cells in culture. Biochem Biophys Res Commun..

[5] Bar-Nur O, Verheul C, Sommer AG, Brumbaugh J, Schwarz BA, Lipchina I (2015). Lineage conversion induced by pluripotency factors involves transient passage through an iPSC stage. Nat Biotechnol.

[6] Bickenbach JR, Chism E (1998). Selection and extended growth of murine epidermal stem cells in culture. Exp Cell Res..

[7] Bilousova G, Chen J, Roop DR (2011). Differentiation of mouse induced pluripotent stem cells into a multipotent keratinocyte lineage. J Invest Dermatol..

[8] Bilousova G, Roop DR (2013). Generation of functional multipotent keratinocytes from mouse induced pluripotent stem cells. Methods Mol Biol..

[9] Brennand KJ, Simone A, Jou J, Gelboin-Burkhart C, Tran N, Sangar S (2011). Modelling schizophrenia using human induced pluripotent stem cells. Nature..

[10] Chen Y, Mistry DS, Sen GL (2014). Highly rapid and efficient conversion of human fibroblasts to keratinocyte-like cells. J Invest Dermatol.

[11] de Lazaro I, Bussy C, Yilmazer A, Jackson MS, Humphreys NE, Kostarelos K (2014). Generation of induced pluripotent stem cells from virus-free in vivo reprogramming of BALB/c mouse liver cells. Biomaterials.

[12] Doulatov S, Daley GQ (2013). Development. A stem cell perspective on cellular engineering. Science.

[13] Gambaro K, Aberdam E, Virolle T, Aberdam D, Rouleau M (2006). BMP-4 induces a Smad-dependent apoptotic cell death of mouse embryonic stem cell-derived neural precursors. Cell Death Differ..

[14] Greaves NS, Iqbal SA, Baguneid M, Bayat A (2013). The role of skin substitutes in the management of chronic cutaneous wounds. Wound Repair Regen..

[15] Gupta MK, Teo AK, Rao TN, Bhatt S, Kleinridders A, Shirakawa J (2015). Excessive cellular proliferation negatively impacts reprogramming efficiency of human fibroblasts. Stem Cells Translat Med.

[16] Hochedlinger K, Plath K (2009). Epigenetic reprogramming and induced pluripotency. Development..

[17] Israel MA, Yuan SH, Bardy C, Reyna SM, Mu Y, Herrera C, Hefferan MP, Van Gorp S, Nazor KL, Boscolo, FS, et al. Probing sporadic and familial Alzheimer’s disease using induced pluripotent stem cells. Nature. 2012;482:216–20. 10.1038/nature10821 PMC3338985 22278060

[18] Itoh M, Kiuru M, Cairo MS, Christiano AM (2011). Generation of keratinocytes from normal and recessive dystrophic epidermolysis bullosa-induced pluripotent stem cells. Proc Natl Acad Sci U S A..

[19] Kelaini S, Cochrane A, Margariti A (2014). Direct reprogramming of adult cells: avoiding the pluripotent state. Stem Cells Cloning..

[20] Lichti U, Anders J, Yuspa SH (2008). Isolation and short-term culture of primary keratinocytes, hair follicle populations and dermal cells from newborn mice and keratinocytes from adult mice for in vitro analysis and for grafting to immunodeficient mice. Nat Protoc..

[21] Lujan E, Chanda S, Ahlenius H, Sudhof TC, Wernig M (2012). Direct conversion of mouse fibroblasts to self-renewing, tripotent neural precursor cells. Proc Natl Acad Sci U S A..

[22] Margariti A, Winkler B, Karamariti E, Zampetaki A, Tsai TN, Baban D (2012). Direct reprogramming of fibroblasts into endothelial cells capable of angiogenesis and re-endothelialization in tissue-engineered vessels. Proc Natl Acad Sci U S A.

[23] Marion RM, Strati K, Li H, Tejera A, Schoeftner S, Ortega S, Serrano M, Blasco, MA. Telomeres acquire embryonic stem cell characteristics in induced pluripotent stem cells. Cell Stem Cell. 2009;4:141–54. 10.1016/j.stem.2008.12.010 19200803

[24] Pang ZP, Yang N, Vierbuchen T, Ostermeier A, Fuentes DR, Yang TQ, Citri A, Sebastiano V, Marro S, Sudhof TC, et al. Induction of human neuronal cells by defined transcription factors. Nature. 2011;476:220–3. 10.1038/nature10202 PMC3159048 21617644

[25] Qian L, Huang Y, Spencer CI, Foley A, Vedantham V, Liu L, Conway SJ, Fu JD, Srivastava D. In vivo reprogramming of murine cardiac fibroblasts into induced cardiomyocytes. Nature. 2012;485:593–8. 10.1038/nature11044 PMC3369107 22522929

[26] Rouhani F, Kumasaka N, de Brito MC, Bradley A, Vallier L, Gaffney D (2014). Genetic background drives transcriptional variation in human induced pluripotent stem cells. PLoS Genet.

[27] Ruiz S, Panopoulos AD, Herrerias A, Bissig KD, Lutz M, Berggren WT (2011). A high proliferation rate is required for cell reprogramming and maintenance of human embryonic stem cell identity. Curr Biol.

[28] Silva J, Barrandon O, Nichols J, Kawaguchi J, Theunissen TW, Smith A (2008). Promotion of reprogramming to ground state pluripotency by signal inhibition. PLoS Biol..

[29] Song K, Nam YJ, Luo X, Qi X, Tan W, Huang GN, Acharya A, Smith CL, Tallquist MD, Neilson EG, et al. Heart repair by reprogramming non-myocytes with cardiac transcription factors. Nature. 2012;485:599–604. 10.1038/nature11139 PMC3367390 22660318

[30] Stadtfeld M, Maherali N, Breault DT, Hochedlinger K (2008). Defining molecular cornerstones during fibroblast to iPS cell reprogramming in mouse. Cell Stem Cell..

[31] Suprynowicz FA, Upadhyay G, Krawczyk E, Kramer SC, Hebert JD, Liu X, Yuan H, Cheluvaraju C, Clapp PW, Boucher Jr RC, et al. Conditionally reprogrammed cells represent a stem-like state of adult epithelial cells. Proc Natl Acad Sci U S A. 2012;109:20035–40. 10.1073/pnas.1213241109 PMC3523865 23169653

[32] Takahashi K, Tanabe K, Ohnuki M, Narita M, Ichisaka T, Tomoda K, Yamanaka S. Induction of pluripotent stem cells from adult human fibroblasts by defined factors. Cell. 2007;131:861–72. 10.1016/j.cell.2007.11.019 18035408

[33] Takahashi K, Yamanaka S (2006). Induction of pluripotent stem cells from mouse embryonic and adult fibroblast cultures by defined factors. Cell..

[34] Tolar J, Xia L, Riddle MJ, Lees CJ, Eide CR, McElmurry RT, Titeux M, Osborn MJ, Lund TC, Hovnanian A, et al. Induced pluripotent stem cells from individuals with recessive dystrophic epidermolysis bullosa. J Invest Dermatol. 2011;131:848–56. 10.1038/jid.2010.346 PMC4151825 21124339

[35] Uitto J (2011). Regenerative medicine for skin diseases: iPS cells to the rescue. J Invest Dermatol..

[36] Vierbuchen T, Ostermeier A, Pang ZP, Kokubu Y, Sudhof TC, Wernig M (2010). Direct conversion of fibroblasts to functional neurons by defined factors. Nature..

[37] Vierbuchen T, Wernig M (2011). Direct lineage conversions: unnatural but useful?. Nat Biotechnol..

[38] Wernig M, Zhao JP, Pruszak J, Hedlund E, Fu D, Soldner F, Broccoli V, Constantine-Paton M, Isacson O, Jaenisch R. Neurons derived from reprogrammed fibroblasts functionally integrate into the fetal brain and improve symptoms of rats with Parkinson’s disease. Proc Natl Acad Sci U S A. 2008;105:5856–61. 10.1073/pnas.0801677105 PMC2311361 18391196

[39] Yilmazer A, de Lazaro I, Bussy C, Kostarelos K (2013). In vivo cell reprogramming towards pluripotency by virus-free overexpression of defined factors. PLoS One..

[40] Yuspa SH, Kilkenny AE, Steinert PM, Roop DR (1989). Expression of murine epidermal differentiation markers is tightly regulated by restricted extracellular calcium concentrations in vitro. J Cell Biol..

